# Abnormalities in Dynamic Brain Activity Caused by Mild Traumatic Brain Injury Are Partially Rescued by the Cannabinoid Type-2 Receptor Inverse Agonist SMM-189

**DOI:** 10.1523/ENEURO.0387-16.2017

**Published:** 2017-08-18

**Authors:** Yu Liu, Samuel S. McAfee, Natalie M. Guley, Nobel Del Mar, Wei Bu, Scott A. Heldt, Marcia G. Honig, Bob M. Moore, Anton Reiner, Detlef H. Heck

**Affiliations:** 1 Department of Anatomy and Neurobiology, University of Tennessee Health Science Center, Memphis, TN 38163; 2 Department of Pharmaceutical Sciences, University of Tennessee Health Science Center, Memphis, TN 38163; 3 Department of Ophthalmology, University of Tennessee Health Science Center, Memphis, TN 38163

**Keywords:** cannabinoid type 2 receptor, Coherence, mild traumatic brain injury, Neuronal oscillations, Phase amplitude coupling

## Abstract

Mild traumatic brain injury (mTBI) can cause severe long-term cognitive and emotional deficits, including impaired memory, depression, and persevering fear, but the neuropathological basis of these deficits is uncertain. As medial prefrontal cortex (mPFC) and hippocampus play important roles in memory and emotion, we used multi-site, multi-electrode recordings of oscillatory neuronal activity in local field potentials (LFPs) in awake, head-fixed mice to determine if the functioning of these regions was abnormal after mTBI, using a closed-skull focal cranial blast model. We evaluated mPFC, hippocampus CA1, and primary somatosensory/visual cortical areas (S1/V1). Although mTBI did not alter the power of oscillations, it did cause increased coherence of θ (4-10 Hz) and β (10-30 Hz) oscillations within mPFC and S1/V1, reduced CA1 sharp-wave ripple (SWR)-evoked LFP activity in mPFC, downshifted SWR frequencies in CA1, and enhanced θ-γ phase-amplitude coupling (PAC) within mPFC. These abnormalities might be linked to the impaired memory, depression, and persevering fear seen after mTBI. Treatment with the cannabinoid type-2 (CB2) receptor inverse agonist SMM-189 has been shown to mitigate functional deficits and neuronal injury after mTBI in mice. We found that SMM-189 also reversed most of the observed neurophysiological abnormalities. This neurophysiological rescue is likely to stem from the previously reported reduction in neuron loss and/or the preservation of neuronal function and connectivity resulting from SMM-189 treatment, which appears to stem from the biasing of microglia from the proinflammatory M1 state to the prohealing M2 state by SMM-189.

## Significance Statement

Using a mouse model, we show that mild traumatic brain injury (mTBI) caused significant abnormalities in oscillatory neuronal activity patterns in CA1 of hippocampus and medial prefrontal cortex (mPFC), two structures strongly implicated in cognitive and emotional processes. The cannabinoid type-2 (CB2) receptor specific inverse agonist SMM-189, which has been shown to reverse behavioral deficits and neuronal loss in the present model of mTBI, also rescued most of the electrophysiological deficits. Taken together, our current and previously published studies suggest that use of CB2 receptor inverse agonists such as SMM-189, which appear to act by biasing microglia from the proinflammatory M1 state to the prohealing M2 state, may be a promising therapeutic approach for mTBI.

## Introduction

Mild traumatic brain injury (mTBI) involving a closed-head injury from a blow, blast, or sudden acceleration/deceleration event is common in civilian, sports and military settings (Miller, 2011a,b; Weinberger, 2011) and can result in cognitive and emotional deficits (Lundin et al., 2006; Malojcic et al., 2008; Niogi et al., 2008; Bryant et al., 2010; Miller, 2011a,b; Weinberger, 2011; Bazarian et al., 2013). The brain pathology associated with mTBI involves diffuse axonal injury (Browne et al., 2011; Johnson et al., 2013; Heldt et al., 2014), but no obvious foci of neuronal loss or other damage (Bazarian et al., 2013). It thus remains uncertain which brain areas are altered so as to produce these deficits and how mTBI affects neuronal activity in brain areas linked to cognition and emotion. The cognitive and emotional deficits typically associated with mTBI strongly implicate the medial prefrontal cortex (mPFC) and the hippocampus, as these two structures are of central importance for cognitive and emotional functions (Bahner et al., 2015; Spellman et al., 2015; Sigurdsson and Duvarci, 2016). The role of the mPFC and hippocampus in cognitive and emotional processes is characterized by oscillatory neuronal activity at specific frequencies, which transiently synchronizes the activity of neurons within and between the structures (Rauch et al., 2006; Sierra-Mercado et al., 2006; Covington et al., 2010; Popa et al., 2010; Maroun, 2013; Russo and Nestler, 2013; Bocchio and Capogna, 2014; Mueller et al., 2014; Zelikowsky et al., 2014). γ Oscillations (30-100 Hz) in these structures are thought to be of critical importance to cognitive processing, and they are often observed to be nested within specific phases of lower frequency δ (1-3 Hz), θ (4-7 Hz), and α frequency oscillations (8-12 Hz) in a phenomenon known as phase-amplitude coupling (PAC). The generation of these functionally relevant patterns of neuronal activity, particularly of oscillations that exhibit coherence between regions and coupling of oscillatory frequencies within a local network of neurons, requires the integrity of network connections and synaptic communication (Buzsáki et al., 2013). Thus, coherence and PAC of oscillatory neuronal activity reflect functional connectivity patterns in larger neuronal networks (Antonakakis et al., 2015). Coherence analyses of MEG and EEG signals in patients have shown that TBI in humans causes deficits in functional connectivity between brain areas (Varotto et al., 2014; Dimitriadis et al., 2015). We analyzed coherence and PAC of oscillations in hippocampal CA1 region (CA1) and mPFC in a mouse model of mTBI to evaluate the whether mTBI would cause similar electrophysiological abnormalities in these areas. We also characterized hippocampal sharp wave ripples and their effect on mPFC local field potential (LFP) responses to further quantify deficits in oscillatory neuronal activity and functional connectivity. Hippocampal sharp-wave ripples (SWRs) have been suggested as a mechanism to redistribute newly encoded memories from the hippocampus to the neocortex (Wierzynski et al., 2009).

The focal cranial air blast model of mTBI used here mimics closed-skull impact, the most common type of TBI (Heldt et al., 2014; Reiner et al., 2014; Guley et al., 2016). The model is characterized by diffuse axonal injury, microglial activation, and a variety of sensory, motor, cognitive, and emotional deficits. In particular, the memory deficits include defective working memory out to eight months post-blast as assessed by spontaneous alternation in an X-maze (our unpublished observations), depression as assessed by tail suspension (Heldt et al., 2014; Reiner et al., 2014), and heightened learned fearfulness as assessed using auditory fear conditioning (Heldt et al., 2014; Reiner et al., 2014).

We performed all electrophysiological measurements in awake, head fixed mice (Bryant et al., 2009), and observed a variety of abnormalities after mTBI. Additionally, we assessed whether the cannabinoid type-2 (CB2) receptor inverse agonist SMM-189 (Presley et al., 2015) rescued any of the mTBI-related abnormalities in oscillatory neuronal activity, coherence and PAC in the mPFC and hippocampus. We chose SMM-189 because previous studies with the same mouse model have shown that the drug rescued mTBI-related neurologic deficits, including depression and fear perseveration reduced neuron loss caused by mTBI (Reiner et al., 2014; Bu et al., 2016).

## Materials and Methods

### Animal care

Thirty-seven adult (more than three to four months) male mice (C57BL/6J) were used in the present study. Mice were housed within a breeding colony at the University of Tennessee Health Science Center animal facilities with 12/12 h light/dark cycles in standard cages with free access to food and water. All animal procedures were performed in accordance with the National Institutes of Health Guide for the Care and Use of Laboratory Animals (2011). Experimental protocols were approved by the University of Tennessee Health Science Center Institutional Animal Care and Use Committee.

### Summary of time course of experimental procedures

(1) Adult mice between three and four months of age received controlled left side air blasts to the closed skull to induce mTBI or received sham treatment as described in (Heldt et al., 2014; Guley et al., 2016).

(2) Beginning at 2 h following the blast or sham treatment, mice received daily injections for two weeks of either drug (SMM-189) or vehicle as described previously (Reiner et al., 2014).

(3) Four to five weeks after the blast or sham treatment, electrophysiological recordings were performed in mPFC, CA1 and S1/V1 in awake, head fixed conditions as described below.

(4) After completion of experiments, recording sites were verified histologically.

### Mild TBI induction and drug/vehicle treatments

In the first experiment, mice were randomly divided into the sham-vehicle (vehicle-treatment only), mTBI-vehicle (blast + vehicle) and mTBI-drug (blast + SMM-189) groups (nine mice in each group). In a second set of experiments, we evaluated the effect of SMM-189 on a separate group of 10 sham-treated mice, randomly assigned to receive SMM-189 or vehicle treatment (five mice in each group).

Mild TBI was induced by a focal closed-head air blast of 50-psi to the left side of the head. Sham-treated mice underwent the same procedures but did not receive an air blast. SMM-189 was administered daily (6 mg/kg injected i.p., dissolved in vehicle; ethanol:cremophor:0.9% saline; 5:5:90; between 0.1 and 0.2 ml, depending on body weight). The first injection was administered within 2 h after mTBI or sham treatment. This mTBI model was developed and validated by Reiner and colleagues (Heldt et al., 2014; Guley et al., 2016), who have also evaluated the efficacy of SMM-189 in this model (Reiner et al., 2014; Bu et al., 2015). We used a blast pressure level of 50-psi, which produces widespread axonal injury and microglial inflammation with minimal mortality (Heldt et al., 2014; Reiner et al., 2014; Guley et al., 2016). Briefly, the overpressure air blast was delivered by a small horizontally mounted air cannon system. Mice were anesthetized with Avertin (400 mg/kg body weight) and secured within a foam rubber sleeve inside a pair of Plexiglas tubes with their targeted head region positioned in the center of a 7.5-mm diameter hole in the outer tube and the air cannon opening positioned 4-5 mm from the hole in the outer tube. This arrangement restricted the blast exposure to a 7.5-mm diameter area on the left side of the mouse cranium between ear and eye. The rest of the mouse was completely shielded from the blast by the Plexiglas tubes. The foam rubber sleeve surrounding the mouse cushioned the nonblast side of the mouse. Afterward, animals recovered on a heating pad. Tylenol (35 mg/ml) was added to the drinking water for 24 h following blast exposure for sham and blast-treated mice.

### Choice of air pressure parameters for mild TBI induction

Previous studies using the same mouse model for mTBI we employed here evaluated the effects of air blast pressures ranging from 0-psi (sham) to 60-psi on three-month-old male C57BL/6 mice (Heldt et al., 2014; Reiner et al., 2014; Bu et al., 2016; Guley et al., 2016). Those studies found that mice subjected to single blasts of 40-psi or less showed no significant motor or behavioral deficits in tests for depression and fear learning and little obvious neuronal damage or axonal injury. By contrast, histologic analysis of mice subjected to a single blast of 50-60 psi revealed axonal injury, neuronal loss, and microglial activation in a variety of brain regions (Heldt et al., 2014; Bu et al., 2016; Guley et al., 2016). These mice were reported to also demonstrate a variety of deficits during the first few months after blast, and most notably for present interest, memory loss (our unpublished observations), increased depression and fear perseveration (Heldt et al., 2014; Guley et al., 2016). To further explore the basis of the deficits with 50- to 60-psi blasts, here we used a blast pressure of 50-psi to examine the effects of mTBI on neuronal activity in mPFC, S1/V1 and CA1, because of the deficits it yields with minimal mortality. We found that mice exposed to 50-psi focal closed-head cranial blasts to the left side of the head showed a variety of deficits in neuronal activity patterns in the left mPFC and hippocampal CA1, which are likely to be contributory to the previously reported behavioral deficits, especially those concerning memory, fearfulness, and mood.

### Treatment groups

In a first set of experiments, we used a 50-psi blast to induce mTBI in two groups of nine mice each. A third group of nine mice in the first set of experiments received sham treatment, i.e., they were exposed to identical procedures with the exception that no air blast was delivered to the cranium. Mice exposed to mTBI-producing blasts were randomly assigned to either an mTBI-vehicle (blast + vehicle) or an mTBI-drug (blast + SMM-189 treatment) group. Sham-treated mice from this group received vehicle injections only.

A separate group of 10 mice was used to investigate the effects of SMM-189 versus vehicle treatment on sham-treated mice. Mice were randomly divided into two groups of 5 mice each which received either daily injections of SMM-189 or vehicle, following the same treatment schedules and procedures used for the first set of experiments.

Because SMM-189 is not soluble in water, it was dissolved in a 0.9% saline vehicle solution containing ethanol and cremophor (90:5:5), both of which are regarded as safe for human use by the Food and Drug Administration. After the completion of vehicle or drug treatment, mice were housed for two more weeks before electrophysiological recordings started.

### Surgery

**Figure 1.**
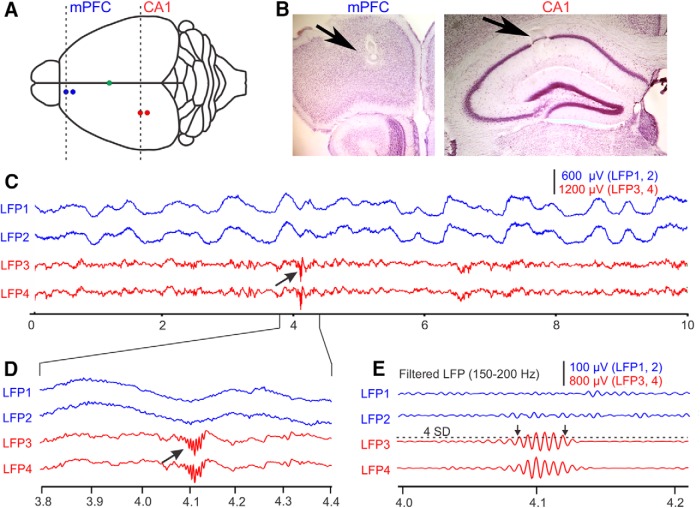
Recording sites and raw data examples of simultaneous recordings of LFPs in the awake mouse mPFC and hippocampal CA1 region. ***A***, Schematic drawing of the top view of a mouse brain. LFPs were simultaneously recorded from the left PFC along the boundary between the frontal association area and prelimbic cortex (blue dots) and S1/V1 transition area (red dots). The electrodes in S1/V1 (red dots) were advanced into the CA1 region of the hippocampus following recording in S1/V1. The green dot represents bregma. Dashed lines represent the coronal sections for verifying recording locations in the mPFC and CA1 in a mouse not included in this study but recorded following identical procedures (see Materials and Methods). ***B***, Electrolytic lesions (arrows) in the mPFC (left) and the CA1 region of hippocampus (right) marking sites from which recordings shown in ***C*** were obtained. ***C***, Examples of raw LFP data recorded at sites indicated in ***A***. LFP1 and LFP2 were recorded from electrodes positioned in the mPFC (blue); LFP3 and LFP4 were recorded from electrodes in the CA1 (red). Arrow points at a characteristic high-frequency ripple activity. Abscissa represents time in seconds. ***D***, An enlarged view of raw LFPs around a hippocampal ripple event (arrow). Same LFP amplitude scale bar as in ***C***. ***E***, High-pass filtered versions of the LFPs in panel ***D*** emphasizes the high-frequency ripple components of CA1 activity. Horizontal dashed line above LFP3 represents the mean filtered LFP amplitude plus 4 SD from a continuous data of 60 s, which was used as a threshold (mean ± 4 SD) for automatic detection of SWR activity in the CA1 region. Left and right arrows mark the beginning and the end of ripple activity, respectively.

Four to five weeks after blast or sham treatments, mice were surgically prepared for awake, head fixed, electrophysiological recordings. Surgical anesthesia was initiated by exposing mice to a mix of 3% isoflurane in oxygen in an incubation chamber. Anesthesia was maintained with 1-2% isoflurane in oxygen during surgery using an Ohio isoflurane vaporizer (Highland Medical Equipment). Rectal temperature was maintained at 37-38˚C with a servo-controlled heat blanket (FHC). To prepare for electrophysiological recordings from the blasted side of the brain, two round skull openings (1.0-1.5 mm in diameter) over the left mPFC and the left hippocampus were made using a dental drill (Microtorque II, RAM Products) without damaging the underlying dura (Fig. 1*A*). Stereotaxic coordinates for the center of the medial prefrontal cortical target area were 0.5 mm lateral to midline and 2.8 mm anterior to bregma. For the hippocampal CA1 target region, coordinates were 2.0 mm lateral to midline and 2.3 mm posterior to bregma. A cylindrical plastic recording chamber (0.45-cm diameter and 8-mm height) was placed over the skull openings and a metal head-post was mounted on the skull for head fixation during experiments. The chamber and head-post were embedded in acrylic cement and anchored to the skull bone using three small skull screws. The chamber was completely filled with triple antibiotic ointment. While still under anesthesia, mice were injected subcutaneously with Carprofen solution (0.05 ml; 50 mg/ml) to alleviate pain. A postsurgical recovery period of 3-4 d was allowed before electrophysiological experiments.

### Electrophysiological experiments

Mice were adapted to the head-fixed position by placing them in the head holder for increasing amounts of time before the first recording session. We recorded from each mouse up to three times but only once per day. Before each recording session, the chambers were cleaned and filled with saline solution. Four extracellular recording electrodes (glass insulated tungsten/platinum; 80 μm in diameter; impedance: 3.5-5.0 MΩ) were used to record LFPs. During experiments, the guiding tubes of a computer-controlled microdrive (Thomas Recording) were lowered into the saline-filled recording chamber to a distance of <1 mm from the dural surface. In the Thomas Recording System, the guiding tubes also serve as reference electrodes and their electrical connection to the brain tissue is established via the saline solution. Two recording electrodes (80 μm in diameter, 350 μm apart) were slowly advanced through the intact dura into the mPFC along the border between the frontal association area and the prelimbic cortex. A second pair of electrodes was advanced into the neocortex directly overlying the hippocampal CA1 region, which is an area that lies at the caudal boundary of the primary somatosensory cortex (S1) and the rostral boundary of the primary visual cortex (V1), and which we thus refer to as S1/V1. After recording from the S1/V1 region of neocortex, the electrodes were lowered into the CA1 proper for subsequent recordings. Statistical comparisons of coherence results from the rostral S1 and caudal V1 recordings sites revealed no significant differences. Therefore, we pooled all data recorded at S1/V1 sites. Since this study focused on alterations in LFP activity, no effort was made to isolate single unit spike activity. The accuracy of electrode penetrations was verified postmortem for all animals by reference to surface maps of the location of cortical areas and hippocampus (Paxinos and Franklin, 2001; Mohajerani et al., 2013). During the recordings from CA1, penetration depth and the occurrence of characteristic SWRs in the LFP signal were used to verify the localization of the electrode tip in the CA1 region (Buzsáki, 2015; Fig. 1*C-E*). Recording depth in the mPFC was estimated based on anatomically confirmed recording sites from recordings in >10 mice that were performed before and were not related to this study. No lesions were made in blast-treated mice included in this study, to minimize tissue damage, which could interfere with subsequent immunohistochemical analysis, which is not part of the current study. However, the depth of electrode placement was again verified for all recordings from sham-treated mice and showed successful placement of electrode tips in the mPFC using the same procedures as for the blast-treated mice. An example of electrolytic lesions marking the recording sites in the mPFC and CA1 is shown in (Fig. 1*B*).

Simultaneous recordings of LFPs from the mPFC and S1/V1 or from the mPFC and hippocampal CA1, with two electrodes placed within each structure, were used to evaluate both the within-structure and the between-structure coherence of LFP oscillations and of an mPFC-CA1 interaction in the form of CA1-SWR evoked LFP-responses in the mPFC. All signals were bandpass filtered at 0.1-200 Hz, digitized at 2 kHz and saved to a hard-disk (CED 1401 and Spike2 software, Cambridge Electronic Design).

### Data analysis

#### LFP coherence

Coherence was calculated for LFP signals recorded at two recording sites within the mPFC, S1/V1 or CA1. We calculated coherence both between the electrodes in a given structure, as well as coherence between structures. For between-structure coherence, we used the more anteriorly located electrodes in each structure for consistency. Recordings consisted of continuous 10-min samples of LFP activity. From the 10-min recordings, five 1-min periods without movement artifacts were selected and exported from Spike2 to a Matlab (Mathworks) file format. Coherence analysis was performed in Matlab (Matlab, R2015a) using custom scripts (Matlab function code: mscohere).

#### Detection of SWR activity in the CA1

For the analysis of hippocampal SWRs, raw LFPs were bandpass filtered at the frequency range known to contain of SWR frequencies (Sullivan et al., 2011; Schlingloff et al., 2014; 150-200 Hz; Fig. 1*E*). The mean amplitude and SD of LFP amplitude fluctuation across each 1 min data block were calculated from the band passed signal. Potential SWR onsets were detected as LFP values larger or smaller than the average LFP value by ± 4 SDs. A minimum of five-ripple waveforms (defined as five equidistant voltage peaks) was required for the detected potential SWR onset to qualify as a true SWR. The end of an SWR was marked as the first LFP voltage that fell within the ± 4 SDs voltage range around the mean LFP, with the following voltage values remaining within this range for 100 ms. Based on this criterion, we treated SWRs separated by at least 100 ms as two distinct SWR events.

#### Time-frequency analysis of SWRs

To examine time-frequency aspects of SWR activity in the CA1 region of the hippocampus, LFPs were analyzed using an open-source software FieldTrip (Oostenveld et al., 2011). Sections of LFP data from 0.6 s before and 0.6 s after SWR onsets were selected for the performance of SWR-aligned time-frequency analysis (FieldTrip function: ft_freqanalysis; method: mtmconvol; tapper: hanning; window size: 0.2 s; step: 1 ms; sample rate: 2000 Hz; frequency rang: 100-220 Hz).

#### PAC analysis of LFP

The same sections of LFP data in mPFC and CA1 used for coherence analysis were also selected for PAC analysis. In particular, the relationships between the phases of LFP oscillations in the δ, θ, and α frequency range (1-12 Hz) and the amplitudes of oscillations in the γ frequency range (30-100 Hz) were evaluated and then compared between groups. The PAC of LFP oscillations was quantified using an open-source Matlab Toolbox written by Angela Onslow (www.cs.bris.ac.uk/Research/MachineLearning/pac), documented previously (Bruns and Eckhorn 2004; Canolty et al., 2006; Osipova et al., 2008; Onslow et al., 2011). Values corresponding to the degree of PAC (i.e., modulation index indicating the extent to which low-frequency phase is related to high-frequency amplitude) were normalized before averaging or grouping within conditions.

#### Statistical analyses

One-way ANOVA in MATLAB Statistics Toolbox (code: anova1) was used to analyze changes in LFP activities between experimental groups (code: multcompare; *post hoc* test: Tukey-Kramer). Unless specified otherwise, figures represent results as mean ± SE.

### Histology

In sham-treated mice, an electrolytic lesion in mPFC and/or CA1 was made by passing an electrical current (10 μA; 12 s) through one of the recording electrodes. Lesions were made at the end of the final experiments, and no electrolytic lesions were made in the S1/V1 region. All animals were deeply anesthetized and intracardially perfused with 0.9% NaCl and followed by 4% paraformaldehyde solution. Brains were removed and fixed in 4% paraformaldehyde solution for a minimum of 24 h. The accuracy of electrode penetrations was verified postmortem for all animals by reference to surface maps of the location of cortical areas and hippocampus (Paxinos and Franklin, 2001; Mohajerani et al., 2013). For animals with electrolytic lesions, the fixed brains were sectioned at 60 μm and mounted onto slides. Light microscopy was used to verify the accurate depth of penetration of the recording electrode in the PFC and the CA1 region of the hippocampus (Fig. 1*B*).

## Results

### Resting-state coherence of LFP oscillation within mPFC, S1/V1, and hippocampal CA1 region

**Figure 2.**
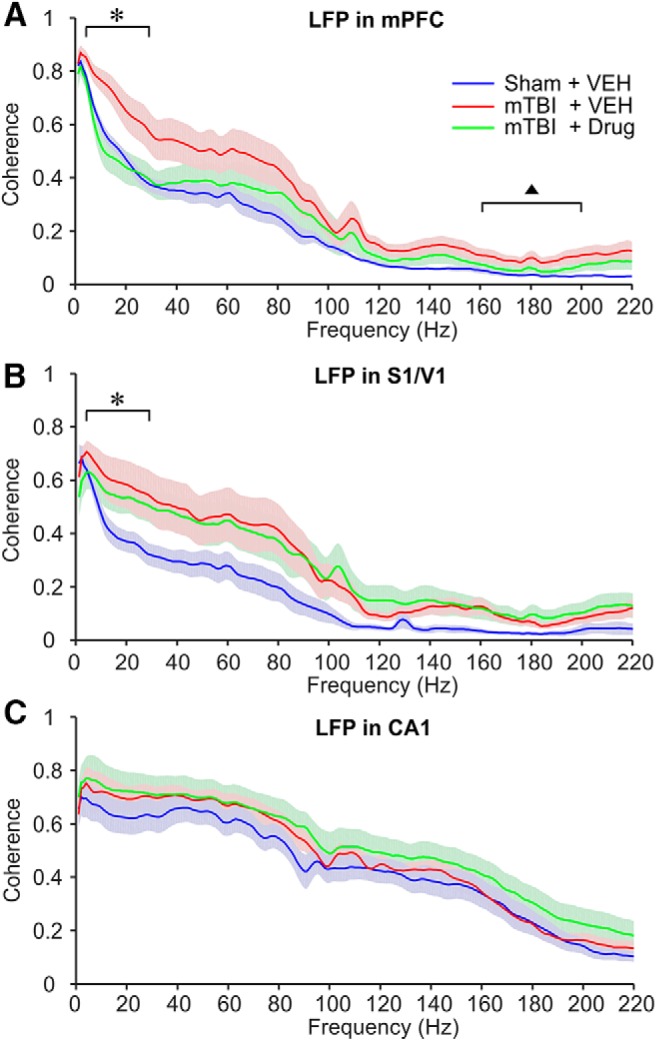
Coherence in the LFP oscillation in mice with mTBI. ***A***, mPFC. *F_(2,24)_ = 5.2474, *p* = 0.0129; sham + VEH versus mTBI + VEH: *p* = 0.0499. ^▲^*F*_(2,24)_ = 3.4297, *p* = 0.0490; sham + VEH versus mTBI + VEH: *p* = 0.0411. ***B***, S1/V1. *F_(2,24)_ = 4.1190, *p* = 0.0337; sham + VEH versus mTBI + VEH: *p* = 0.0326. ***C***, Hippocampal CA1 region. The same color codes are used for all panels. Data were expressed as mean ± SE. Comparisons of mean coherence between 4 and 30 Hz were conducted using one-way ANOVA (*post hoc* test: Tukey-Kramer). VEH, vehicle.

Four to five weeks after left side 50-psi or sham blast, LFP recordings were obtained from sites within the left mPFC, hippocampal CA1 region or the S1/V1 cortex (Fig. 1). Analysis of the oscillation power spectra for each brain region revealed no significant differences between treatment groups (one-way ANOVA; data not shown). Mild TBI did, however, cause a significant elevation of LFP oscillation coupling (coherence) across the two mPFC recording sites, significantly so in the low (1-30 Hz) and high (150-200 Hz) frequency oscillation ranges in mTBI vehicle-treated mice (Fig. 2*A*), compared to the sham vehicle-treated mice. A similar pattern of elevated coherence was observed across the two recording electrodes in S1/V1 in mTBI mice compared to the sham vehicle-treated mice, but only the elevation in the low-frequency coherence (1-30 Hz) reached statistical significance (Fig. 2*B*). Mild TBI caused no changes in LFP coherence in CA1 (Fig. 2*C*). Treatment with SMM-189 normalized LFP coherence in the mPFC (Fig. 2*A*) but not in S1/V1 (Fig. 2*B*). SMM-189 treatment had no effect on within-CA1 LFP coherence compared to the sham vehicle-treated mice (Fig. 2*C*), neither increasing nor lowering it.

### Resting-state coherence of LFP oscillation between mPFC and S1/V1 and between mPFC and CA1

Coherence of oscillatory LFP activity between mPFC and S1/V1 or between mPFC and CA1 was not significantly altered by mTBI. There was also no significant effect of drug treatment on between-region coherence (one-way ANOVA used for both analyses; data not shown).

### Frequency and power of SWR activity in CA1

**Figure 3.**
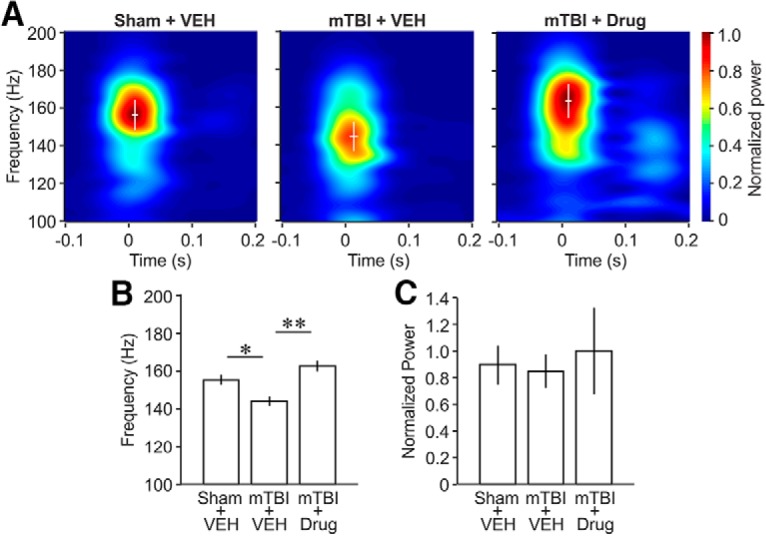
Time-frequency analysis of ripple activity in the hippocampal CA1 region. ***A***, Time-frequency mapping of LFP around CA1 ripples. Data are aligned on the onset of ripple activity (at time 0 s). Color represents frequency power normalized to the maximal power in the three groups. The white cross that is centered at the peak-frequency-power represents mean ± SD of the peak-power times (abscissa) and peak frequencies (ordinate) of ripple activities. ***B***, ***C***, Comparisons of peak frequency ***B*** and frequency power of ripple activity ***C***. Data are expressed as mean ± SE. One-way ANOVA (*post hoc* test: Tukey-Kramer) for peak frequency in ***B***: *F*_(2,24)_ = 12.6401, *p* = 0.0463; *p = 0.0159; **p = 0.0001. VEH, vehicle.

We asked whether mTBI affected the peak frequency or power of the rapid oscillations characterizing the SWRs in CA1 and whether SMM-189 treatment would normalize any such potential change in SWR activity. Time-frequency analysis of SWR oscillations revealed a significant drop in the peak SWR oscillation frequency to ∼145 Hz in 50-psi blast vehicle-treated compared to ∼155 Hz in sham blast vehicle-treated mice (Fig. 3*A*,*B*). The SWR peak frequency in 50-psi blast drug-treated mice, by contrast, was statistically no different from in sham blast vehicle-treated mice, suggesting that SMM-189 treatment rescued SWR peak frequency after mTBI (Fig. 3*A*,*B*). There were no significant differences in the relative power of oscillatory activity within the ripple-frequency band (150-200 Hz) among the three groups, i.e., the amplitude of the SWRs was not affected by mTBI or SMM-189 treatment (Fig. 3*C*).

### Change in SWR-associated LFP

**Figure 4.**
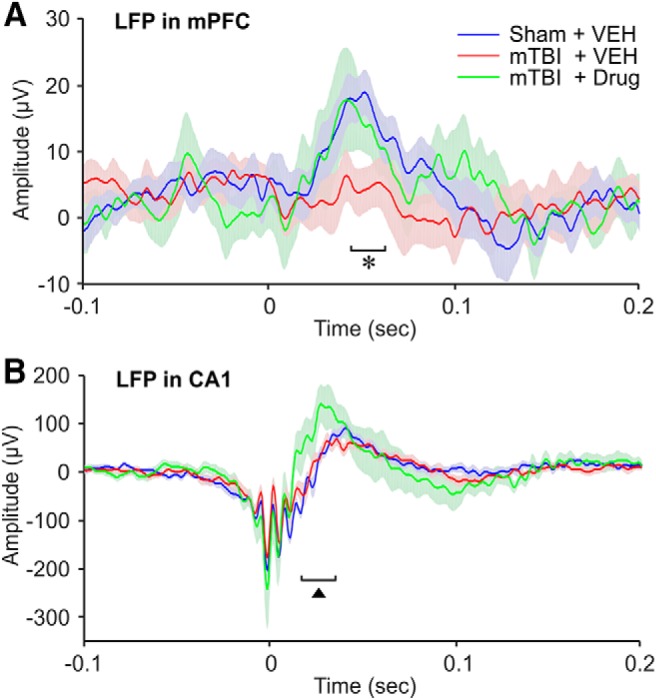
Changes in the amplitude of LFP in the mPFC during hippocampal ripple activities. ***A***, ***B***, LFPs recorded in the mPFC and hippocampal CA1 region, respectively. Data are aligned on the onset of the hippocampal ripples (*t* = 0 s). One-way ANOVA (*post hoc* test: Tukey-Kramer): *F_(2,24)_ = 3.5031, *p* = 0.0463; sham + VEH versus mTBI + VEH: *p* = 0.0456. ^▲^*F*_(2,24)_ = 4.0177, *p* = 0.0313; sham + VEH versus mTBI + drug: *p* = 0.0489. VEH, vehicle.

Although the coherence of oscillations between CA1 and mPFC was not altered by mild TBI, analysis of SWRs showed that TBI did affect communication between these two regions. Hippocampal SWRs have been shown to alter neuronal activity in the PFC, and this modulation has been implicated in the transfer of memory content from hippocampal to cortical storage (Wierzynski et al., 2009). We examined the effect of mTBI on mPFC responses to the occurrence of spontaneous SWRs in CA1 by analyzing the average LFP response in the mPFC aligned on SWR onset (Fig. 4). In the sham blast group, SWRs in CA1 evoked a positive LFP deflection in the mPFC, which reached its peak amplitude around 50 ms after SWR onset. The SWR-evoked response in the mPFC was significantly reduced in mTBI-vehicle-treated mice, and treatment of mTBI mice with SMM-189 rescued the mPFC response to CA1 SWRs (Fig. 4*A*). The average amplitude of SWRs in CA1 was itself, however, not altered in mTBI-vehicle treated compared to sham-treated mice (as noted above). However, mTBI drug-treated mice showed a significant SWR amplitude increase in CA1 during the late positive phase of the SWR (Fig. 4*B*). This late-phase increase in amplitude was also reflected in time-frequency analysis in the form of a temporary increase in power in the 5- to 10-Hz range (data not shown).

### LFP oscillation PAC of δ/θ/α phase with γ amplitude

**Figure 5.**
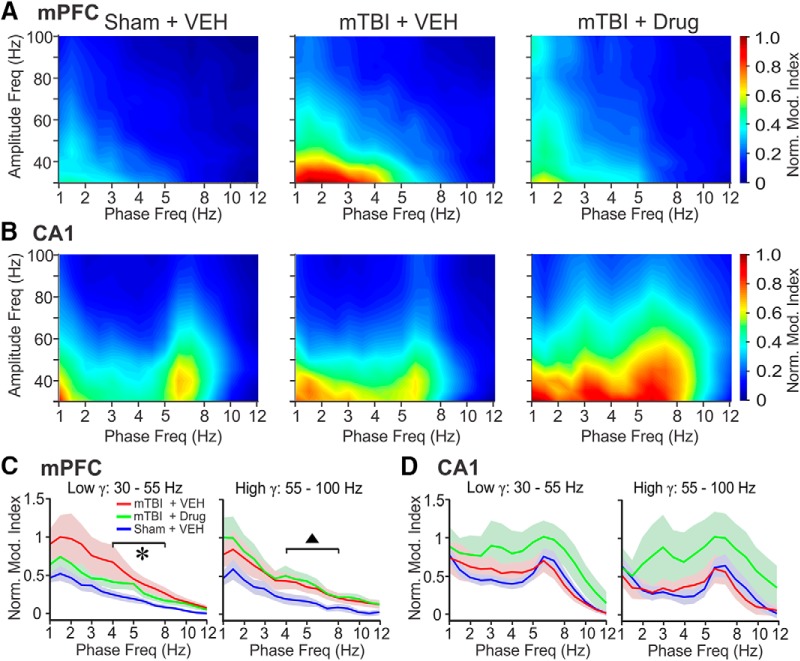
θ Oscillatory phase modulates γ-band LFP activity in mice with mTBI. ***A***, PAC mapping of LFP activity in the mPFC. PAC values (color-coded) are normalized to the maximum value found within the three experimental groups within each region. ***B***, PAC mapping of LFP activity in CA1. The same method for data-normalization as in ***A*** was used. ***C***, Line-graph representations of PAC in the mPFC for each experimental condition, shown separately for the low γ (low γ, left panel) and high γ (high γ, right panel) frequency ranges. Data are expressed as mean ± SE. One-way ANOVA (*post hoc* test: Tukey-Kramer): *F_(2,24)_ = 2.8977, *p* = 0.0746; sham + VEH versus mTBI + VEH: *p* = 0.0604. ^▲^*F*_(2,24)_ = 3.0115, *p* = 0.0681; sham + VEH versus mTBI + drug: *p* = 0.0759. ***D***, Same analysis as in panel ***C*** but for the hippocampal CA1 region. Same data legends as in ***C***. Norm. Mod. Index, normalized modulation index; VEH, vehicle.

PAC is a phenomenon in which the amplitude of γ band oscillations increases selectively during a specific phase of a simultaneously occurring slower oscillation. Cross-frequency PAC has been observed in multiple brain structures, including the mPFC and CA1 (Li et al., 2012; Ito et al., 2013; Lisman and Jensen, 2013; Vaidya and Johnston, 2013; Roux and Uhlhaas, 2014), and has been implicated in cognitive processes such as context-dependent reward learning (Tort et al., 2009). We performed a PAC analysis separately for the amplitude of low (30-55 Hz) and high (55-100 Hz) γ frequency ranges relative to the phases of frequencies in the δ, θ, and α range (1-12 Hz). In the mPFC of 50-psi blast mice that received vehicle injections, θ to γ PAC was significantly increased for both low and high γ frequencies, compared to sham mice (Fig. 5*A*,*C*). By contrast, in CA1 PAC between θ (∼5-8 Hz) and γ was similar in blast and sham mice (Fig. 5*B*,*D*). Following drug treatment, neither mPFC nor CA1 PAC were significantly different from that in sham-vehicle mice for low-frequency γ (Fig. 5*B*-*D*), but SMM-189 did not correct PAC abnormality for high γ seen in the vehicle-treated 50-psi mice.

### Effects of SMM-189 on neuronal activity in healthy, sham-treated mice

**Figure 6.**
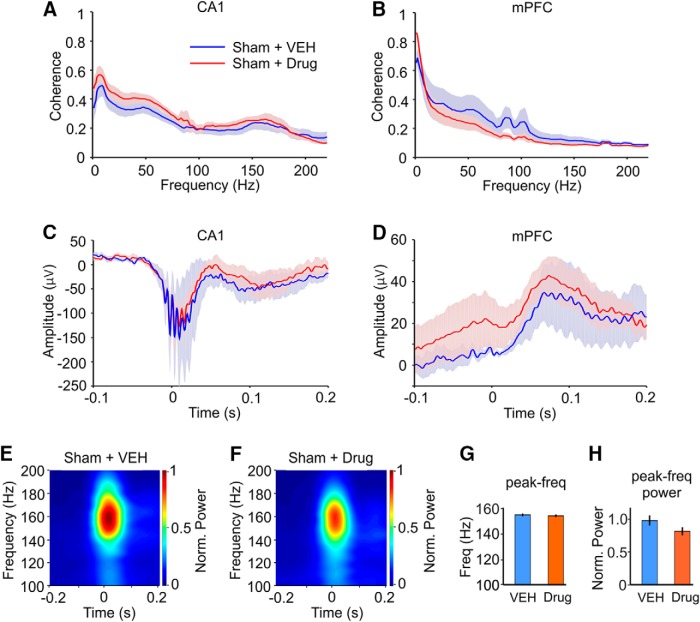
Effects of vehicle (VEH, blue traces) and SMM-189 (drug, red traces) treatment on neuronal activity in sham-treated control mice. ***A***, Coherence of LFP in hippocampal CA1 region (CA1) in drug and vehicle-treated sham mice. ***B***, Coherence of LFP in the mPFC. ***C***, Average SWR wave form in CA1 in drug and vehicle-treated mice. ***D***, Average LFP responses in the mPFC aligned with SWR onset in drug and vehicle-treated mice. ***E***, ***F***, Time-frequency mapping of SWR activity in CA1 in drug and vehicle-treated mice. Time 0 corresponds to SWR onset. Pseudocolors represent frequency power normalized to the maximum power (Norm. Power) within both groups. ***G***, ***H***, Quantitative representation and comparison of peak frequency (***G***) and normalized peak power (***H***) of SWR activity shown in panels ***E***, ***F***. Statistical comparison (one-way ANOVA) of all quantitative endpoints between drug versus vehicle-treated mice revealed no differences between the two groups in any of the measures.

Ten mice were treated with sham blasts and randomly assigned to receive daily SMM-189 (*n* = 5) or vehicle (*n* = 5) injections for two weeks, i.e., the same treatment regimes as the blast-treated mTBI mice. Comparison of drug and vehicle-treated mice showed that in healthy mice SMM-189 treatment did not alter any of the electrophysiological measures we analyzed. LFP coherence within the CA1 and PFC was the same in drug and vehicle-treated mice (Fig. 6*A*,*B*), as were hippocampal SWR wave form, SWR-aligned mPFC LFP responses (Fig. 6*C*,*D*) and the SWR peak frequency and power (Fig. 6*E-H*). The main findings are summarized in Table 1.

**Table 1.** Summary of effects of SMM-189 on LFP and hippocampal SWR activity in mice with mTBI

	CA1			mPFC			S1/V1		
	Sham + drug	mTBI + VEH	mTBI + drug	Sham + drug	mTBI + VEH	mTBI + drug	Sham + drug	mTBI + VEH	mTBI + drug
LFP power spectrum	Same as sham + VEH	Same as sham + VEH	Same as sham + VEH	Same as sham + VEH	Same as sham + VEH	Same as sham + VEH	Same as sham + VEH	Same as sham + VEH	Same as sham + VEH
LFP coherence within area	Same as sham + VEH	↑	Same as sham + VEH	Same as sham + VEH	Same as sham + VEH	Same as sham + VEH	Same as sham + VEH	↑	↑
LFP coherence with CA1	N/A	N/A	N/A	Same as sham + VEH	Same as sham + VEH	Same as sham + VEH	N/A	N/A	N/A
LFP coherence with S1/V1	N/A	N/A	N/A	Same as sham + VEH	Same as sham + VEH	Same as sham + VEH	N/A	N/A	N/A
SWR frequency	Same as sham + VEH	↓	Same as sham + VEH	N/A	N/A	N/A	N/A	N/A	N/A
SWR relative frequency power	Same as sham + VEH	Same as sham + VEH	Same as sham + VEH	N/A	N/A	N/A	N/A	N/A	N/A
SWR-associated LFP amplitude	Same as sham + VEH	Same as sham + VEH	↑	Same as sham + VEH	↓	Same as sham + VEH	N/A	N/A	N/A
θ-γ coupling	N/A	Same as sham + VEH	Same as sham + VEH	N/A	↑	Same as sham + VEH	N/A	N/A	N/A

CA1, hippocampal CA1 region; N/A, not applicable; VEH, vehicle; ↑, increase; ↓ , decrease.

## Discussion

We examined cortical and hippocampal oscillatory neuronal activity patterns in the mPFC and CA1 of awake, head-fixed mice to determine if mTBI produces abnormalities in these brain regions and thereby to determine if dysfunction in these structures contributes to the resulting cognitive and neuropsychiatric deficits. Previous studies have shown that mTBI in the closed-head, focal air blast mouse model used here causes diffuse axonal damage and neuron death, and a variety of sensory, motor, cognitive, and emotional deficits (the latter including fearfulness and depression), as also observed in mTBI patients (Heldt et al., 2014; Bu et al., 2016; Guley et al., 2016). In the present study, we observed several neurophysiological abnormalities in oscillatory activity linked to the role of mPFC and CA1 in cognition, mood and affect, including in coherence and PAC of oscillatory neuronal activity, mPFC responses to hippocampal SWRs, and the peak frequency of SWRs. Specifically, the coherence of oscillatory activity was increased across the δ to β frequency range (2-30 Hz) after mTBI in the mPFC, but not in hippocampal CA1. PAC between θ phase and γ amplitude was also significantly increased in the mPFC, but not in CA1 after mTBI.

Our electrophysiological recordings were all made on the left side of the brain, the side that was targeted by the blast. Previous studies have shown that neuron loss is restricted to the left side for the basolateral amygdala, whereas it is similar for the right and left sides of cerebral cortex and striatum (Bu et al., 2016). Further experimental work is needed to determine if electrophysiological abnormalities in mPFC and CA1 are similar on the contralateral side or less severe

The rescue of these electrophysiological abnormalities with SMM-189 treatment, which has been shown in previous studies to rescue mTBI behavioral deficits, is consistent with the interpretation that the abnormalities in oscillatory behavior in mPFC and CA1 may play a role in the behavioral deficits. SMM-189 is known to target CB2 receptors on microglia and causes a bias in microglial activation from the proinflammatory (M1) to the prohealing (M2) state (Presley et al., 2015). Thus, the most likely mechanism behind the rescue of electrophysiological deficits we observed here is the rescue of neuron death and synapse loss by the modulatory effect of SMM-189 on neuroinflammatory responses. By contrast, SMM-189 treatment had no effect on any measures of neuronal activity in healthy, sham-treated mice, suggesting that the SMM-189 benefit in mTBI did not stem from some effect that proved compensatory for the mTBI abnormalities. The implications of these findings are considered in more detail below.

## Role of oscillatory activity in brain function

Oscillations in LFPs represent the coordinated rhythmic activation of large populations of synaptic inputs to the neurons at the site of LFP recording (Buzsáki et al., 2012). This oscillatory activity influences the timing of neuronal activation within local ensembles of neurons, thus affecting communication between each such ensemble and other neuronal populations to which it projects, for example in distant target regions. Coherence of oscillations within a given brain region also are of note, since the phase locking of activity within a region can affect the granularity of the output from that region. Consistent with its importance in neuronal communication, oscillatory neuronal activity, including dynamic changes in the coherence of oscillations (Fries, 2015) and PAC (Canolty et al., 2006; Tort et al., 2009), has been widely linked to cognitive brain functions, including attention (Fries et al., 2001; Laufs et al., 2003; Tallon-Baudry, 2004; Fries, 2015), perception (Engel et al., 2001; Tallon-Baudry, 2003), decision making (Gould et al., 2012; Wyart et al., 2012; Nácher et al., 2013), problem solving (Sheth et al., 2009), and memory (Marshall et al., 2006; Tort et al., 2009; Chauvette et al., 2012; Anderson et al., 2014). Abnormalities in these neuronal oscillatory patterns have been observed in a variety of brain disorders and are regarded as revealing neuronal dysfunction that underlies cognitive and emotional deficits, including in mTBI (Leon-Carrion et al., 2012; Haneef et al., 2013; Bailey et al., 2014; Dimitriadis et al., 2015). In particular, abnormalities in oscillatory activity in the mPFC have been strongly linked to depression and deficits in fear extinction, both in humans (López et al., 2014; Olbrich et al., 2014) and rodents (Milad and Quirk, 2002; Sierra-Mercado et al., 2006). With regard to the latter, Quirk and colleagues have shown that fear extinction is an active process requiring an intact and normally functioning mPFC, and that fear perseveration ensues otherwise (Milad and Quirk, 2002). Additionally, studies in humans have linked the modulation of γ oscillation power in the mPFC to fear extinction (Mueller et al., 2014). Perseverance of fearful memories and depression are characteristic findings in patients suffering from mTBI (Kennedy et al., 2007; Bombardier et al., 2010; Miller, 2011a; Reiner et al., 2014), and could thus stem from mPFC dysfunction.

### Coherence abnormalities in our studies

With these considerations, the abnormalities we observed in mPFC coherence following mTBI may be contributors to deficits in working memory in a spontaneous cross-maze alternation task (our unpublished observations), as well as to the depression and fearfulness reported with this model (Heldt et al., 2014). The reversal of both behavioral deficits (Reiner et al., 2014) and coherence abnormalities in multiple frequency ranges with SMM-189 further supports the view that the electrophysiological dysfunction in mPFC reflects functional changes that contribute to the cognitive and emotional deficits associated with mTBI.

The temporal coordination of the phases of oscillatory neuronal activity requires the integrity of axonal projections and of synaptic connections (Buzsáki et al., 2013). Diffuse, widespread axonal damage and neuron death are characteristic features of mTBI (Browne et al., 2011; Johnson et al., 2013; Lafrenaye et al., 2015; Bu et al., 2016) and may be key factors underlying the oscillatory abnormalities observed here. Although mTBI might be expected to result in a reduction of coherence strength if there is loss of network connectivity or synaptic inputs from other structures, we found a significant increase in coherence in the mPFC of mTBI mice across a low-frequency range (1-30 Hz) covering δ, θ, α, and β bands, and in a high frequency range (150-200 Hz). It is unclear how mTBI would cause increased coherence. Coherence studies in rodent models of Parkinson’s disease have shown that the loss of dopamine causes increases in cortico-striatal coherence (Sharott et al., 2005). Whether loss of dopaminergic neurons and/or their connections is responsible for increased mPFC coherence after mTBI in mice remains to be shown. Alternatively, or in addition, the loss of excitatory extrinsic inputs from other cortical regions or from thalamus may cause mPFC to default to heightened coherence driven by intrinsic connectivity.

### SWR abnormalities in our studies

The hippocampal network generates characteristic SWR activity which has been shown to be critically involved in memory consolidation and memory retrieval in mice (Malvache et al., 2016; Nicole et al., 2016; van de Ven et al., 2016), rats (Jadhav et al., 2012; Singer et al., 2013; Pfeiffer and Foster, 2015; Maingret et al., 2016; Papale et al., 2016; O'Neill et al., 2017; Rothschild et al., 2017; Wu et al., 2017), and nonhuman primates (Logothetis et al., 2012; Ramirez-Villegas et al., 2015; Leonard and Hoffman, 2017). We found that mTBI caused a reduction in the average frequency of hippocampal SWRs and eliminated SWR-evoked responses in the mPFC. Given the crucial role of hippocampal SWRs and their transmission to the mPFC for long-term memory functions (Wierzynski et al., 2009), deficits in SWR frequency and SWR-evoked responses in the mPFC may be causally related to memory deficits associated with mTBI (Lundin et al., 2006; Sim et al., 2012).

### PAC abnormalities in our studies

We also found that coupling between the phase of θ band oscillations and the amplitude of γ oscillations in the mPFC was increased in mTBI mice. Treatment of mTBI mice with SMM-189 partially rescued this PAC abnormality in the lower γ frequency range (30-55 Hz) but not in the high γ frequency range (55-100 Hz). It has been suggested that PAC coordinates neuronal communication within and across brain regions related to information processing (Canolty and Knight, 2010). The strength of PAC within the mPFC correlates with correct versus incorrect decisions in a spatial working memory task in rats (Li et al., 2012), supporting the link between PAC and cognitive processes. Currently, however, it is uncertain how the strength of PAC is controlled and we can thus only speculate how mTBI may increase PAC. One possibility is that reduced axonal connectivity after TBI decreases the influence of some modulatory input that normally maintains PAC within a specific range. It is also likely that diffuse neuronal death from mTBI results in changes to regional balances of excitatory and inhibitory synaptic activity, as this balance is known to underlie the amplitude and frequency of γ oscillations (Economo and White, 2012; Brunel and Wang, 2013). Whether and to what degree these or other mechanisms contribute to the PAC abnormality remains to be determined.

## Mild TBI: mechanisms of injury and rescue with SMM-189

Mild TBI involves both primary damage from the compressive, tensile and shear forces during the closed-head injury and secondary damage due to neuroinflammatory responses set in motion by the trauma (Joseph et al., 2015; Lafrenaye et al., 2015; Werner and Stevens, 2015; Loane and Kumar, 2016). CB2 receptors are primarily expressed by microglia in the brain after mTBI (Elliott et al., 2011; Donat et al., 2014; Lopez-Rodriguez et al., 2016). We have previously shown that the CB2 receptor inverse agonist SMM-189 mitigates behavioral deficits and neuropathology caused by mTBI (Reiner et al., 2014). More recently we reported that SMM-189 significantly rescues mTBI induced neuron death in the neocortex, basal ganglia and the basolateral amygdala (Bu et al., 2016). These findings, together with the well-known involvement of microglia in neuron death and the pruning and plasticity of synapses (Brown and Vilalta, 2015; Hong et al., 2016; Loane and Kumar, 2016), provide a possible mechanism of benefit for a drug that acts predominantly on microglia to rescue the currently observed mTBI-related electrophysiological deficits. Coherence and PAC of oscillatory neuronal activity reflect functional connectivity patterns in larger neuronal networks (Antonakakis et al., 2015). Thus, a deficit in network connectivity through neuron death and/or synapse loss following mTBI would inevitably lead to abnormalities in coherence and functional connectivity. Thus, it is likely that the ability of SMM-189 to rescue the electrophysiological abnormalities reported here is ultimately based on the prevention or reduction of neuron death and synapse loss. Which activity patterns are rescued and which are not, and in which specific brain regions rescue occurs, is likely to depend on the severity of damage sustained in a specific area and possible differences between types of neurons in susceptibility to mTBI-related cell death.

Interestingly, SMM-189 treatment did not rescue the coherence deficit in S1/V1 following mTBI. Other than a general loss of network connectivity, we currently have no definitive understanding of the neuropathology and/or cellular changes underlying the current coherence abnormalities. SMM-189 treatment rescues neuron death in the neocortex after mTBI, preventing about half of an overall 20% loss of cortical neurons (Bu et al., 2016), but whether the benefit of SMM-189 is less for the S1/V1 region than for other parts of the neocortex or whether damage in S1/V1 is more severe than in other areas has yet been determined.

A therapeutic potential of CB2 receptor inverse agonists has been proposed for several years (Lunn et al., 2008), but studies of their benefits for brain activity and behavior in neurodegenerative diseases have been scant. The SMM-189 structural class of tri-aryl compounds shows good blood-brain barrier penetration (Fujinaga et al., 2010) and Presley et al. (2015) have shown that SMM-189 binds selectively to the CB2 receptor. Further, SMM-189 has been shown to convert human microglia from the proinflammatory M1 phenotype to the prohealing M2 phenotype (Reiner et al., 2014; Presley et al., 2015), suggesting that the beneficial effects of SMM-189 after mTBI are linked to the alteration of microglial activation state so that microglia act to minimize loss and increase rescue. The previously reported rescue of axonal damage (Reiner et al., 2014) and the rescue of neuron death (Bu et al., 2016) in mTBI mice by SMM-189 treatment are likely to be key factors in the rescue of the diverse mTBI-related electrophysiological deficits we observed with SMM-189. Although the enrichment of microglia in CB2 receptors compared to other brain cell types (Benito et al., 2003; Stella, 2010; Baek et al., 2013) suggests them to be the primary site of action for SMM-189 benefit, we cannot rule out the possibility that SMM-189 benefit involved a neuronal action, as some neuronal localization of CB2 receptors has been reported (Stempel et al., 2016). Nonetheless, as SMM-189 treatment was limited to two weeks after TBI and thus had ended several weeks before our recordings, it is clear the SMM-189 benefit is enduring and not merely palliative in nature.

## Summary

In summary, our findings suggest that mTBI impairs the functioning of mPFC and CA1, structures that are widely implicated in memory, mood and affect. Our studies show that post-mTBI abnormalities in mPFC and CA1 involve neuronal mechanisms implicated in the precise spatial and temporal coordination of neuronal activity within and between brain structures. Our observations were made in awake, head-fixed mice, which most closely corresponds to resting-state recordings in human studies. Resting-state measurements reliably indicate abnormalities in brain function, as shown by studies on such illnesses as Alzheimer’s disease, schizophrenia and autism (Pollonini et al., 2010; Hsiao et al., 2013; Kam et al., 2013) or normal aging (Salami et al., 2014). Thus, resting-state measures of γ power modulation, for example, could be explored for the development of EEG-based diagnostic tools to determine the severity, progression, and/or treatment success in mTBI patients. Although the various electrophysiological abnormalities we observed in the resting-state have been linked to memory, mood and affect, studies in awake behaving mice will be needed to determine if behavior-related activity patterns and associated behaviors are similarly rescued by SMM-189 treatment. Finally, our current results reinforce prior findings on the significant benefits of SMM-189 treatment for mTBI in mice (Reiner et al., 2014; Bu et al., 2016). Of note, we found that SMM-189 does not alter the brain activity in healthy, sham-treated mice. Our findings, combined with those earlier results suggest that drugs like SMM-189, which quell the adverse effects of M1 microglial activation and promote their beneficial M2 phenotype via a targeting of microglial CB2 receptors (Presley et al., 2015), represent a promising treatment strategy for mTBI.

## Synthesis

Reviewing Editor: Jun Chen, University of Pittsburgh Decisions are customarily a result of the Reviewing Editor and the peer reviewers coming together and discussing their recommendations until a consensus is reached. When revisions are invited, a fact-based synthesis statement explaining their decision and outlining what is needed to prepare a revision will be listed below. The following reviewer(s) agreed to reveal their identity: Coleen Atkins, Shan Ping Yu.

Both reviewers are positive about your submission. They felt your results are interesting and significant. However, each of them raised some major and some minor concerns about your study that need to be addressed when you submit a revision of the manuscript.

Below are the specific comments from the two reviewers:

Reviewer #1

This manuscript is a very interesting study investigating the coherence of oscillations between the hippocampus, prefrontal cortex and primary somatosensory/visual cortical areas after TBI. In addition, the investigators determined if a cannabinoid receptor 2 inverse agonist, SMM-189, would improve alterations in these oscillations. This is a novel approach for understanding the pathophysiology of TBI, and incorporates a therapeutic which is being actively studied as a lead candidate for TBI treatment. The study is narrow in scope, but the data do reveal effects of TBI on coherence within the prefrontal cortex and S1/V1 regions of the cortex. In addition, sharp wave ripple peak frequency in the hippocampus CA1 region was affected by TBI. Treatment with the cannabinoid receptor 2 inverse agonist treatment rescued some, but not all of these changes in oscillatory activity. Overall, the manuscript is clear and the data are intriguing. There are some major concerns that need to be addressed.

1. The title is misleading, since not all of the abnormalities in oscillations were rescued by the cannabinoid type 2 receptor inverse agonist. Also, the effects of SMM-189 may have been indirect, and not affecting oscillations, but rather the underlying neuronal death that contributes to the oscillations.

2. Why were sham animals treated with SMM-189 not included in the study? This is a key control that should have been included.

3. The rationale for testing SMM-189 on oscillatory patterns and coherence was not well stated in the introduction. In addition, the potential mechanisms of how SMM-189 rescued the coherence of LFP in the prefrontal cortex as well as the sharp wave ripple amplitude needs to be expanded upon in the discussion. In particular, a discussion of how cannabinoid type 2 receptor can influence neural oscillations should be included.

4. To isolate the ripple frequency band, the LFP was band-passed filtered at 150 - 200 Hz. This may have been too narrow as ripples have been characterized to occur outside of this range. This should be addressed in the methods section.

5. The statistical analysis description in the methods section is incomplete. It does not include how the two-way ANOVA analysis was conducted. The two-way ANOVA analysis should also be described in full in the results section, stating whether the interaction and main effects were significance, and include the F value with degrees of freedom.

6. Figure 2: A two-way ANOVA would have been a more appropriate analysis for this data set. How did the authors interpret the effects of injury and drug?

6. Figure 4: More information is needed in the methods to describe the calculations used to determine power at the ripple frequency band, i.e. sampling rate, fast Fourier transform size, percent overlap and filter type.

7. Figure 5: The phase of the theta oscillation that was coupled to gamma oscillations should be specified.

8. The discussion should address in greater detail how the pathology of this brain injury model resulted in alterations in these particular oscillations.

Minor comments:

1. The first paragraph of the results is rationale for the study. This should be moved to the introduction.

2. Line 378 states that sharp wave ripples only occur in the hippocampus. This should be revised since it has been reported that sharp wave ripples also occur in the medial prefrontal cortex.

3. Line 355 states that high gamma is 150 - 200 Hz. This is incorrect.

4. Minor typographical errors were found throughout the manuscript.

Reviewer #2

The present study describes various electrophysiological abnormalities caused by mild traumatic brain injury using a mouse model of focal air blast. The authors conducted simultaneous recording of local field potential in and between three different brain regions: the prefrontal cortex, the hippocampus, and the neocortex, and found deviations from the normal, healthy animals in several metrics. Most of the abnormalities were alleviated by the chronic treatment with a cannabinoid receptor antagonist beforehand, consistent with previous findings on the effects of the same drug.

Major comments:

The results of the statistical tests need be described with more rigor. Throughout the text, please provide Ns and what they represent (i.e. number of animals or number of events), statistical value (i.e. F) together with degrees of freedom, and actual p (except for Fig. 2, Fig. 3, and Fig 5, where *p<0.05 is more appropriate, I think). Also please specify which post-hoc test was used and its associated values. ANOVA itself does not provide statistical difference in a pairwise fashion, which is reported in the manuscript nonetheless.

Please consider to re-order the figures so that Fig. 4 comes before Fig. 3 and Fig. 3B comes before Fig. 3A, as I think it is more logical to describe the hippocampal ripples first and then describe its downstream reflection in the PFC.

What';s happening in the non-blasted, right side? Are there similar abnormalities or no abnormalities? If repeating experiments is not permissible, please speculate in Discussion.

Discussion is disproportionally long (Results: ~3 pages vs. Discussion: ~6 pages) and off the topic, in my opinion. For examples, I did not see a good connection with current study and references about dopaminergic cells, which is not mentioned at all up to this point. Please consider to reduce some of the content in Discussion.

Figs. 3B and 4A are the same data in different representations. Can you make some connections between the two? For example, did the significant difference observed in the waveform (the overshoot after ripple with the drug) appear in the spectrogram? I gauge this will increase power in very low frequency, say 10 Hz, which is outside the shown range of the spectrogram.

Minor comments:

Title and page 2, line 29, and other places: I do not think the word ‘novel’ is appropriate as the effects of SMM-189 have been reported in various, related studies cited in this manuscript.

Page 5, line 108: Please specify how the animals were anesthetized.

Page 6, line 125: Please specify concentration of SMM-189 in the solution and/or volume you injected. One cannot claim a chemical is safe without specifying dosage. The cocktail contains as much alcohol as beer and, of course, drinking too much beer is not safe to our health.

Page 7, line 150: Each animal was recorded up to 3 times. These are acute recordings, causing damage to the brain each time. Is there any difference in results between them?

Page 7, lines 155 and 156: ‘The stainless steel guiding...’ The sentence is awkward/grammatically wrong. Please revise.

Page 7, line 168: ‘For some animals...’ Please specify exactly how many animals went through this process.

Page 10, line 239, 240, and 241: Inconsistent font.

Page 10, line 241: Please provide results of ANOVA in numbers.

Page 11, line 253: ‘significantly’ Please specify what test was used and its results.

Page 13, line 303: Omission ‘In the present study, observed...’ -> ‘In the present study, we observed...’

Page 15, line 344: Typo ‘Tly’ (probably ‘previously’).

Page 18, line 431: Typo ‘os’ -> ‘of’

Page 30, line 702: Typo ‘indicatedin’ -> ‘indicated in’

Page 30, lines 714 and 715: ‘One-way ANOVA’. Please provide results in numbers. Specify the post-hoc test and its results.

Page 31, line 724: Please describe how power was normalized.

Page 31, line 725: ‘standard deviation of the onset-time (abscissa)’ How is this possible? If the onset time was defined as 0 as described right above, then onset time of all the recordings should be all 0, by definition, and thus there shouldn't be any deviation.

Figs. 2, 3, and 5C: The way statistical significance is indicated (bars above and below traces) was confusing to me. Can you try a different way, perhaps to add an asterisk right above or below each significant data point?

Figs. 4A, 5A, and 5B: Is this from a single event? Averaged event in one animal? Or average of 9 animals in each group?

Fig. 4A: Add label (‘Normalized power’) to the color chart.

Figs. 5A and B: Add labels to the color charts.
